# The Journal of Comparative Medicine and Surgery

**Published:** 1887-01

**Authors:** 


THE JOURNAL OF COMPARATIVE MEDICINE AND SURGERY.—Since Dr. Hummel took hold of this Journal as publisher there has been a complete revolution in its management and the mechanical get-up. It is greatly improved in the latter respect, while the scope of its work has been made broader; and the field, cultivated in detail, more thoroughly worked, and altogether it has assumed a more scientific aspect. The October number comes to us illustrated with colored plates and wood-cuts, and an engraved portrait of Thomas Walley, M. R. C. V. S. The list of contributors embraces the names of a number of workers, already distinguished in their several branches of investigation, such as Frank S. Billings, Drs. James Law, F. R. C. V. S., E. C. Spitzka, M. D. and others. It is indeed gratifying, to see this too mueh neglected branch of Medical Science being represented by such a journal under such excellent management. Dr. A. L. Hummel, 1217 Filbert Street, Philadelphia, publisher. Drs. W. A. Conklin and R. S. Huidekopers, editors. Quarterly at $2 a year. It is the only Journal published on comparative medicine and surgery.

